# Chicken hepatomegaly and splenomegaly associated with novel subgroup J avian leukosis virus infection

**DOI:** 10.1186/s12917-022-03139-1

**Published:** 2022-01-13

**Authors:** Moru Xu, Fusen Hang, Kun Qian, Hongxia Shao, Jianqiang Ye, Aijian Qin

**Affiliations:** 1 Ministry of Education Key Lab for Avian Preventive Medicine, Yangzhou University, No.12 East Wenhui Road, Yangzhou, Jiangsu 225009 P.R. China; 2 Jiangsu Co-innovation Center for Prevention and Control of Important Animal Infectious Diseases and Zoonoses, No.12 East Wenhui Road, Yangzhou, Jiangsu 225009 P.R. China; 3 Joint International Research Laboratory of Agriculture and Agri-Product Safety, the Ministry of Education of China, Jiangsu, 225009 P.R. China

**Keywords:** Hepatomegaly and splenomegaly, novel ALV-J, layer chicken

## Abstract

**Background:**

Subgroup J avian leukosis virus (ALV-J) is an oncovirus which can induce multiple types of tumors in chicken. In this report, we found novel ALV-J infection is closely associated with serious hepatomegaly and splenomegaly in chicken.

**Case presentation:**

The layer chickens from six flocks in Jiangsu province, China, showed serious hemoperitoneum, hepatomegaly and splenomegaly. Histopathological results indicated focal lymphocytic infiltration, cell edema and congestion in the liver, atrophy and depletion of lymphocyte in the spleen. Tumor cells were not detected in all the organs. avian hepatitis E virus (aHEV), which is thought to be the cause of a very similar disease, big liver and spleen disease (BLS), was not detected. Other viruses causing tumors or liver damage including Marek’s disease virus (MDV), reticuloendotheliosis virus (REV), fowl adenovirus (FAdV) and chicken infectious anemia virus (CIAV) were also proved negative by either PCR or RT-PCR. However, we did detect ALV-J in those chickens using PCR. Only novel ALV-J strains were efficiently isolated from these chicken livers.

**Conclusions:**

This is the first report that chicken hepatomegaly and splenomegaly disease was closely associated with novel ALV-J, highlighting the importance of ALV-J eradication program in China.

**Supplementary Information:**

The online version contains supplementary material available at 10.1186/s12917-022-03139-1.

## Background

Avian leukosis virus (ALV) belongs to the retroviral family associated with multiple types of tumors, including lymphoid leukosis, myelocytoma, hemangioma and some other malignant tumors. Currently, ALV can be divided into 11 subgroups based on its envelope protein and cross neutralization patterns [1]. Among these subgroups, subgroup J of ALV (ALV-J) has made huge economic loss to the poultry industry globally. ALV-J infection mainly causes malignant proliferation of hematopoietic cells, myeloid leukemia, and hemangioma in chickens [1]. To date, ALV-J had spread to broiler chicken, layer chicken and many indigenous chicken breeds subsequently [2–4]. Many studies found that the genome sequences of ALV-J isolates and their clinical symptoms were diverse due to their different host range [5, 6]. Although the eradication program has been applied for more than 10 years in China, ALV-J still persists in the farms across the nation, particularly in the indigenous chicken breeds, posing new challenges.

Notably, ALV-J is evolving in a very fast speed due to its high inherent variation. Recently, novel ALV-J strains with large mutations or deletion in genome have been isolated [7]. In addition to mutation or deletion, recombination among different ALV subgroups or strains has also been frequently reported [8, 9]. A novel ALV strain JS15SG01 with the recombination of the ALV-K, ALV-E and ALV-J, which could even invade and injury brain tissue, has been recently identified [10]. The emerge of such novel ALV strains in China poses the risk for the magnification of the disease pattern caused by ALV infection.

In this study, we reported the detection and isolation of novel ALV-J from chickens with hepatomegaly and splenomegaly, and analyzed the molecular characteristics of two ALV-J isolates from these chickens. These results should help us better understand the impact of ALV-J infection to the poultry industry.

## Case presentation

### Case history, gross and histopathological findings

**Fig. 1:**
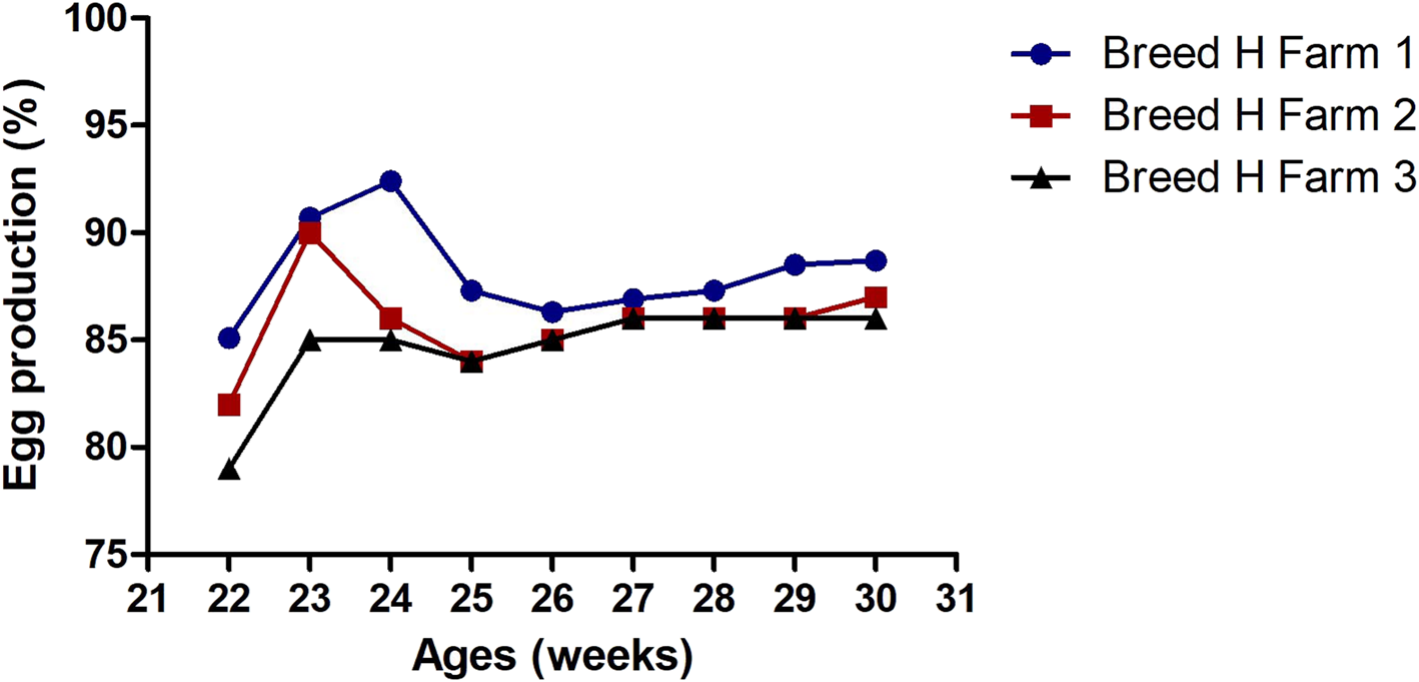
Egg production rates of three farms

The cases with hepatomegaly and splenomegaly were observed in six layer chicken farms in Jiangsu province, China, during February, 2021. This syndrome was firstly observed when those chickens were about 100-day old. When chickens grew to the laying period, the mortality rate of chickens increased while laying rate decreased largely compared with the previous healthy chickens. Three chicken flocks of Breed H (Hy-line grey) with the average age of 33 weeks showed 4.3%, 4.8% and 4.2% of the cumulative mortality rate while three chicken flocks of Breed R (Roman grey) with an average age of 23 weeks demonstrated 1.5%, 0.9% and 0.7% of the cumulative mortality rate, respectively. These chickens showed growth retardation, depression, pale combs and wattles. The laying rate of Breed H chicken in three farms remained below 86% (average) for more than 10 weeks (Fig. 1). The laying rate of Breed R chicken was 10% in the 22^nd^ week and 51% in the 23^rd^ week, significantly lower than that in the healthy counterparts (Data not shown).

**Fig. 2:**
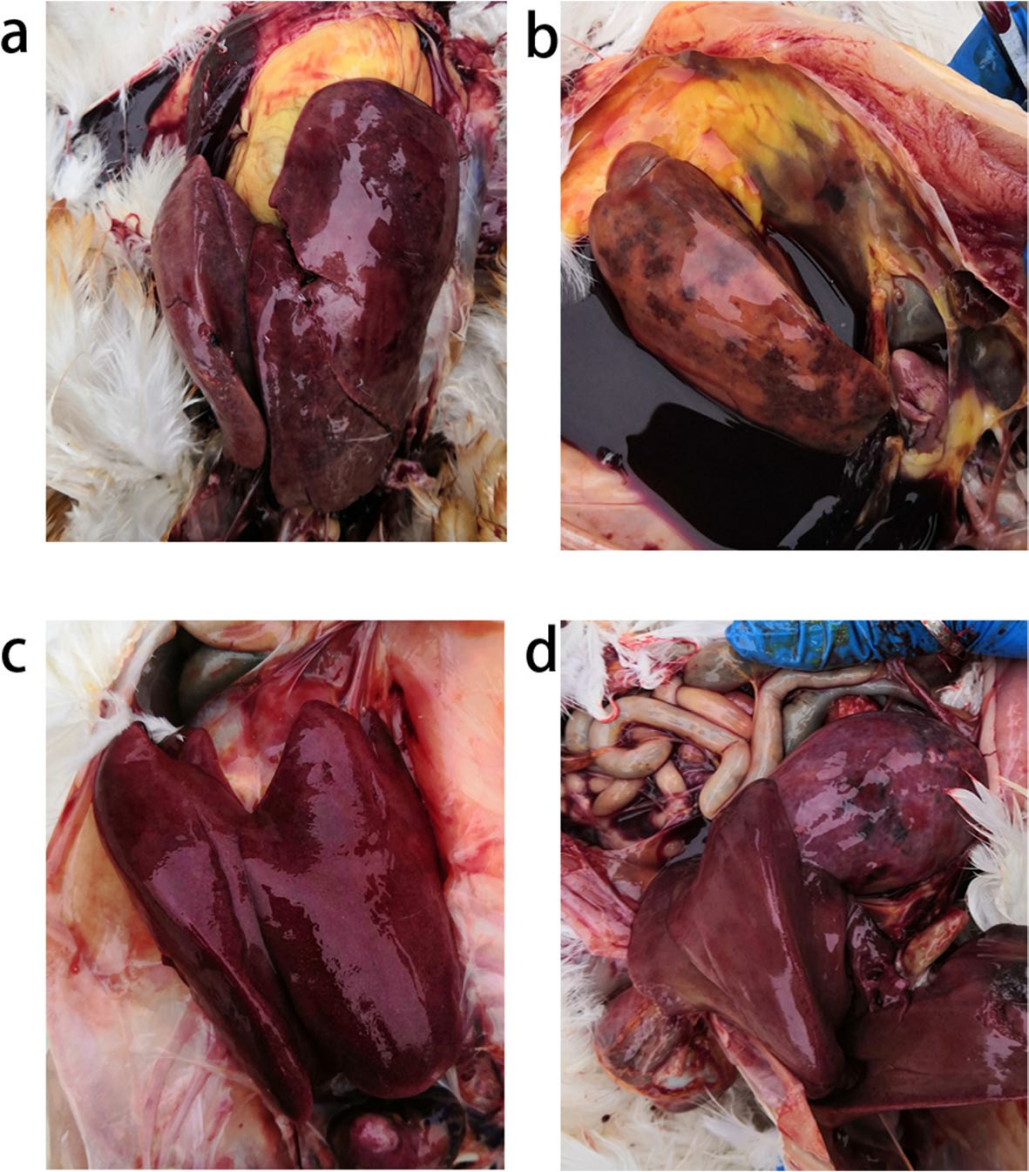
Clinical manifestation of chickens in autopsy. (a) Chickens from Breed R had enlarged livers, with red foci on the surface. (b) Chickens from Breed H had enlarged livers, serious hemoperitoneum was also presented. (c) Chickens from Breed H had enlarged and fragile livers. (d) Chickens from Breed R had mottled and enlarged spleens.

Anatomic analysis showed that most of chickens had enlarged liver, twice to three times bigger than normal ones (Fig. 2a/b/c). According to the farmers, hepatic subcapsular hemorrhage, hepatic rupture along with hemoperitoneum were seen in around 70% of the dead birds. The livers were soft, mottled and fragile, containing many miliary nodules, hemorrhagic spots, stippled with red, yellow foci on the surface (Fig. 2a/b/c). The spleen was also mottled and enlarged, from mildly to severely (Fig. 2d).

**Fig. 3:**
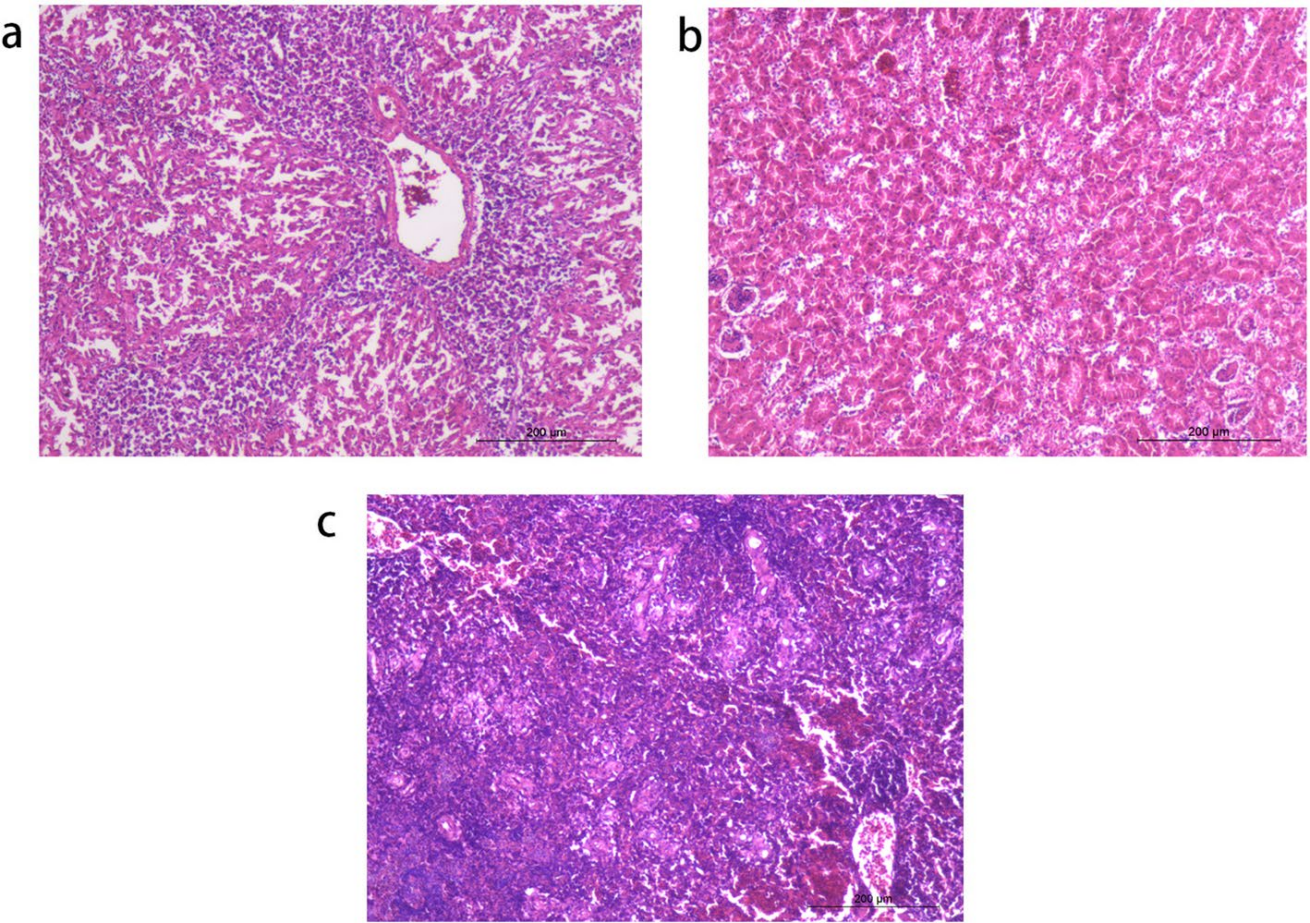
Histological examination of tissue samples. (a) Focal lymphocytic infiltration of heterophils and mononuclear inflammatory cells in the liver. Cell edema were also found in some liver cells. (b) Renal congestion was found in the kidney. (c) Congestion, atrophy and depletion of lymphocyte were detected in the spleen.

Histopathologic images acquired through Olympus BX41 microscope and corresponding software cellSens Entry (Olympus, Japan) further demonstrated that the chicken livers showed focal lymphocytic infiltration of heterophils and mononuclear inflammatory cells (Fig. 3a). Cell edema, congestion and accumulation of some homogenous eosinophilic material, amyloid in the interstitium were also found in some liver cells. However, necrosis was not found in hepatocytes. Renal congestion and renal tubular dilatation were found in the kidney (Fig. 3b), while atrophy and depletion of lymphocyte were detected in the spleen (Fig. 3c).

### Viral detection and isolation

**Fig. 4:**
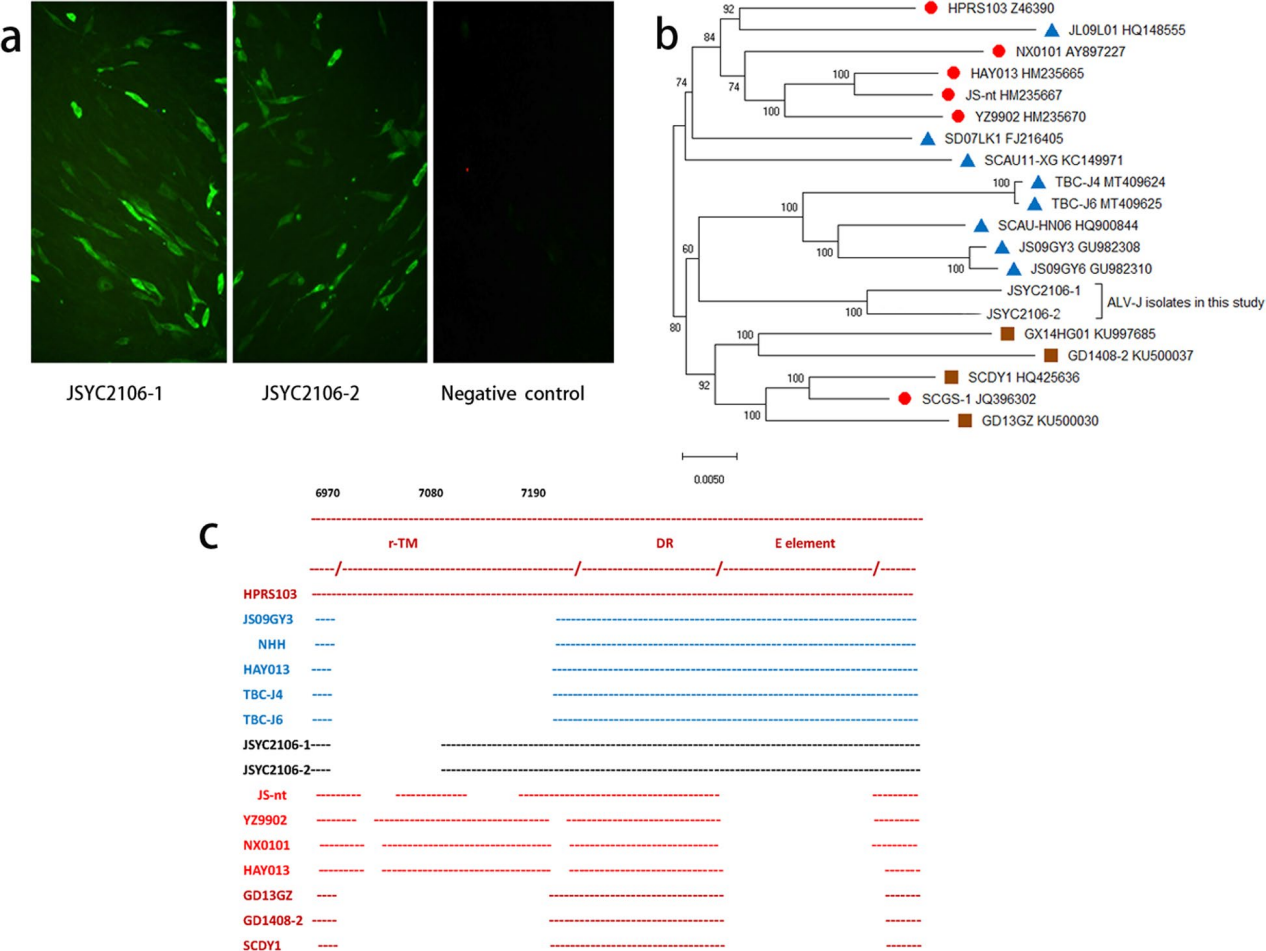
Viral isolation and proviral genome analysis of ALV-J. (a) Detection of isolated ALV-J strain through IFA using monoclonal antibody JE9 against ALV-J. (b) Phylogenetic tree of whole genome of ALV-J strains in this study. Triangles represent layer chicken isolates, circles represent broiler isolates, while squares represent indigenous chicken isolates. (c) Analysis of 3’UTR of ALV-J strains in this study. Blue represents layer chicken isolates, red represents broiler isolates, while brown represents indigenous chicken isolates.

Genomic DNA of six chicken livers with hepatomegaly and splenomegaly was extracted by proteinase K digestion, phenol and chloroform extraction. Using multiple-PCR primers (PF/AR/BR/JR/KR) for ALV, it was found that ALV-J was positive in all the liver samples tested. However, other viruses with potential hepatic damage were not detected, including MDV, REV, CIAV and FAdV (Table S1). RNA of these livers was also extracted for detecting aHEV. After reverse transcription, two nested sets of primers were used for amplifying the ORF2 of aHEV [11], while the results were also negative. To further confirm the ALV-J infection in these samples, DF-1 cells were used to isolate the ALV-J. Two tissue samples (one from Breed H and another from Breed R) were grinded, filtered to make supernatant and inoculated into DF-1 cells. 7 days post infection, the cells were then tested with indirect immunofluorescence assay (IFA) and p27 antigen capture ELISA kits [12]. As described in Fig. 4a, two ALV-J strains were efficiently isolated, designated as JSYC2106-1 and JSYC2106-2, respectively. The whole proviral genomic sequences of the two ALV-J isolates were amplified using 3 pairs of primers (F1/R1, F2/R2 and F3/R3) as previously described [13].

Phylogenetic analysis showed that the whole genome of ALV-J strains JSYC2106-1 and JSYC2106-2 were clustered into the branch of layer chicken ALV-J isolates (Fig. 3b). Homology of the genome of the two strains were 96.3-97.2% with that of the layer chicken ALV-J isolates previously identified in China. Analysis of the *env* gene also showed that the two strains were closer to layer chicken isolates, having the identity of 90.3-91.7% with broiler chicken isolates, 91.1-92.3% with indigenous chicken isolates and 93.4-94.6% with layer chicken isolates, respectively (data not shown). Then, 3’UTR of different ALV-J strains were also compared. As described in Fig. 3c, different ALV-J strains have different deletions in 3’UTR. The two isolates in this study had a deletion of 130bp in redundant transmembrane (r-TM) and carried a complete E element, which is unique among other isolates.

## Discussion and conclusions

In our case, chickens were manifested with the symptom of enlarged liver and spleen, decreased egg production, together with lymphocyte infiltration in the portal vein periphlebitis. These signs are closely related with the description of BLS, which was caused by aHEV. Initially, we suspected whether the chickens were subjecting to aHEV infection. However, those clinical symptoms and lymphocyte infiltration are not specific in aHEV-affected chickens, they can also be observed in some other viral-caused diseases. Meanwhile, although we found edema, congestion and homogenous eosinophilic material in the liver cells, the necrosis was not seen in the histopathology, which was contradict with aHEV infection [14]. Together with the results of PCR and RT-PCR, we believe that ALV-J ought to be strongly associated with this syndrome.

In addition to this case, similar reports had occurred in many farms around China. Previously, we had detected the chickens with the same symptom like this case from different farms in Shandong Province and Guangxi Province (data not shown). Notably, only ALV-J was found in all these samples. Therefore, although we did not perform experiments *in vivo* to directly confirm that ALV-J contributes to hepatomegaly and splenomegaly, ALV-J infection should be closely associated with this syndrome. It deserves our attention that we did not find myelocytoma infiltration in the liver, indicating that hepatomegaly and splenomegaly was not caused by tumor cells. ALV-J might affect chicken liver and spleen through unknown mechanisms.

Previous studies about the tissue tropism of the ALV-J prototype strain HPRS-103 indicated that ALV-J can replicate well in most tissues. Although it does not directly induce tumors in some tissues, like liver and smooth muscle, ALV-J can alter the biological activities of the infected tissues [15]. Our previous research found that ALV-J replicated much faster in Leghorn male hepatoma (LMH) chicken liver cells than traditional DF-1 cells possible due to the high expression level of ALV-J receptor Na^+^/H^+^ exchanger type 1 (NHE1) in LMH cells [16]. It is also found that ALV-J can spontaneously and effectively induce the expression of NHE1 gene in primary chicken liver cells [17]. Both above indicate that the chicken liver is a perfect target for ALV-J infection. Further research in future is required to explore the relationship between ALV-J and liver cells.

Since it was first found in UK in 1989, ALV-J had undergone several significant mutations, together with different symptoms in clinic. ALV-J displays a high level of genetic variation and recombination, which posing the potential for the generation of novel strains with altered phenotypes in antigenicity, tissue tropism, host range and pathogenesis. Within the genomic RNA of ALV-J, *env* gene and 3’UTR are the most variable. Meanwhile, the two parts with mutations can greatly affect the viral pathogenicity. The *env* gene encodes the Env protein, which forms the outer layer of the virion and triggers the viral infection. Two highly variable regions in Env, hypervariable region 1 (hr1) and hypervariable region 2 (hr2), are responsible for the ligand–receptor interaction directly [18], while variable region 3 (vr3) recognizes the specific receptor [14]. The two ALV-J strains isolated in this study belong to the branch of layer chicken ALV-J isolates. However, JSYC2106-1 and JSYC2106-2 show only 90.3%-94.6% homology with other ALV-J strains in *env* gene.

r-TM and E element in the 3’UTR are two important factors which can affect the clinical symptoms directly. The r-TM is considered to be not a necessity for virus replication, but is linked to the pathogenicity of ALV-J in layer chickens [19]. E element is also dispensable for ALV-J, but it is closely related to the oncogenicity and viral replication *in vivo* [20]. E element also contributes to virus-encoded microRNAs [21]. Some early isolates, with a tact r-TM but without the E element, usually cause myeloma in broiler chicken. However, the layer chicken ALV-J isolates with a deletion of 205-bp in the r-TM and all E element can induce hemangioma. The two ALV-J strains JSYC2106-1 and JSYC2106-2 isolated here carry a complete E element, but with a deletion of 130bp in r-TM. It is possible that the two ALV-J strains with the unique 3’UTR might be as the intermediate between broiler and layer chicken ALV-J isolates. However, the pathogenesis of the two ALV-J isolates need to be further tested *in vivo*.

In summary, this report documented the detection and isolation of novel ALV-J in chicken flocks with the disease of hepatomegaly and splenomegaly, highlighting the closely relationship between ALV-J infection and chicken hepatomegaly and splenomegaly, and the significance of the ALV-J eradication program.

## Supplementary Information

**Additional file 1:.** Primers used for PCR and sequencing

## Data Availability

The materials during the study are available from the corresponding author on reasonable request. The genome sequences of two isolates (JSYC2106-1 and JSYC2106-2) are available in GenBank, with the accession numbers OL799231 and OL799232 respectively.

## Abbreviations

ALV-J: Subgroup J avian leukosis virus
aHEV: Avian hepatitis E virus
BLS: Big liver and spleen disease
MDV: Marek’s disease virus
REV: Reticuloendotheliosis virus
FAdV: Fowl adenovirus
CIAV: Chicken infectious anemia virus
PCR: Polymerase chain reaction
RT-PCR: Reverse transcriptase polymerase chain reaction
IFA: Immunofluorescence assay
ELISA: Enzyme-linked immunosorbent assay
