# Supplementary material for: Chicken hepatomegaly and splenomegaly associated with novel subgroup J avian leukosis virus infection
Source: BMC Vet Res. 2022 Jan 13;18:32. doi: 10.1186/s12917-022-03139-1 (PMC8756617; doi:10.1186/s12917-022-03139-1)
Supplement: Supplementary file 1 — Additional file 1:. Primers used for PCR and sequencing [file 12917_2022_3139_MOESM1_ESM.docx]

**Table S1** Primers used for PCR and sequencing

| **Primer** | **Sequence** | **Length (bp)** |
| --- | --- | --- |
| aHEV-F1 | TCGCCT(C)GGTAAT(C)ACA(T)AATGC | 278 |
| aHEV-R1 | GCGTTC(G)CCG(C)ACAGGT(C)CGGCC |  |
| aHEV-F2 | ACA(T)AATGCT(C)AGGGTCACCCG | 242 |
| aHEV-R2 | ATGTACTGA(G)CCA(G)CTG(C)GCCGC |  |
| PF | GGATGAGGTGACTAAGAAAG | 567 |
| AR | GAAAGGGAGGATTGTCTAAGGAG |  |
| BR | GAACCCAACAGTTGTAGTTCTGAT | 417 |
| JR | GGTGAGGTCGCTGACTGTAGACT | 195 |
| KR | TGTGCGTGTGCACCCGGTCT | 742 |
| F1 | TGTAGTGTTATGCAATACTCTT | 2554 |
| R1 | GCATGGGAATCCCCCTCCTA |  |
| F2 | CGAATTCCCAGCGAAAATCT | 3221 |
| R2 | CTTGATCATCCTTTTGGGTGATGT |  |
| F3 | AGGTCGACCCCCGGTTAAGATACGAAT | 2715 |
| R3 | TGAAGCCATCCGCTTCATGCAGGT |  |
| MDV-F | TGCGTGCGTGGTTATTATTTC | 144 |
| MDV-R | AATGGTGAGGTCGCTGACTGT |  |
| CIAV-F | AATGAACGCTCTCCAGAAG | 580 |
| CIAV-R | AGCGGATAGTCATAGTAGAT |  |
| REV-F | ATGAAGACGGGCCTAA | 402 |
| REV-R | AAAGGGGAGGCTAAGA |  |
| FADV-F | GTACTTCCACTACGACTTCCTGCGAAA | 521 |
| FADV-R | GTACGGAAGTAAGCCATAGCTAGCACGT |  |
